# Decrease in membrane fluidity and traction force induced by silica-coated magnetic nanoparticles

**DOI:** 10.1186/s12951-020-00765-5

**Published:** 2021-01-11

**Authors:** Tae Hwan Shin, Abdurazak Aman Ketebo, Da Yeon Lee, Seungah Lee, Seong Ho Kang, Shaherin Basith, Balachandran Manavalan, Do Hyeon Kwon, Sungsu Park, Gwang Lee

**Affiliations:** 1 Department of Physiology, Ajou University School of Medicine, Suwon, 16499 Republic of Korea; 2 School of Mechanical Engineering, Sungkyunkwan University, Suwon, 16419 Republic of Korea; 3 Department of Applied Chemistry and Institute of Natural Sciences, Kyung Hee University, Yongin-si, 17104 Republic of Korea; 4 Department of Molecular Science and Technology, Ajou University, Suwon, 16499 Republic of Korea

**Keywords:** Cell movement, Membrane fluidity, Micropillar, Silica-coated magnetic nanoparticles, Traction force

## Abstract

**Background:**

Nanoparticles are being increasingly used in biomedical applications owing to their unique physical and chemical properties and small size. However, their biophysical assessment and evaluation of side-effects remain challenging. We addressed this issue by investigating the effects of silica-coated magnetic nanoparticles containing rhodamine B isothiocyanate [MNPs@SiO_2_(RITC)] on biophysical aspects, such as membrane fluidity and traction force of human embryonic kidney 293 (HEK293) cells. We further extended our understanding on the biophysical effects of nanoparticles on cells using a combination of metabolic profiling and transcriptomic network analysis.

**Results:**

Overdose (1.0 μg/µL) treatment with MNPs@SiO_2_(RITC) induced lipid peroxidation and decreased membrane fluidity in HEK293 cells. In addition, HEK293 cells were morphologically shrunk, and their aspect ratio was significantly decreased. We found that each traction force (measured in micropillar) was increased, thereby increasing the total traction force in MNPs@SiO_2_(RITC)-treated HEK293 cells. Due to the reduction in membrane fluidity and elevation of traction force, the velocity of cell movement was also significantly decreased. Moreover, intracellular level of adenosine triphosphate (ATP) was also decreased in a dose-dependent manner upon treatment with MNPs@SiO_2_(RITC). To understand these biophysical changes in cells, we analysed the transcriptome and metabolic profiles and generated a metabotranscriptomics network, which revealed relationships among peroxidation of lipids, focal adhesion, cell movement, and related genes and metabolites. Furthermore, in silico prediction of the network showed increment in the peroxidation of lipids and suppression of focal adhesion and cell movement.

**Conclusion:**

Taken together, our results demonstrated that overdose of MNPs@SiO_2_(RITC) impairs cellular movement, followed by changes in the biophysical properties of cells, thus highlighting the need for biophysical assessment of nanoparticle-induced side-effects. 
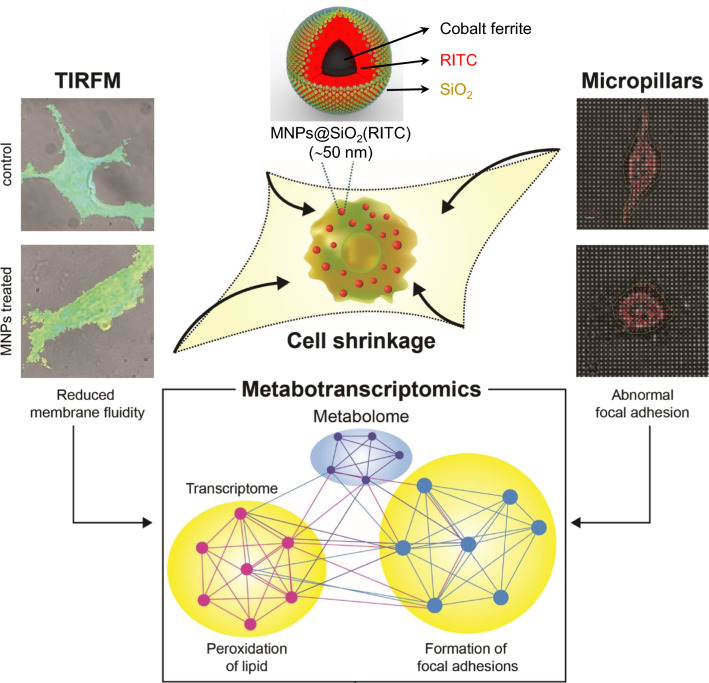

## Background

The use of nanoparticles for diagnostic and therapeutic purposes has been rapidly increasing in medicine. However, as the small size of nanoparticles is known to enable their cellular entry and accumulation, this could potentially cause cellular dysfunction [1–4]. Moreover, compared with bulk materials, nanoparticles are known to be more reactive and owing to their higher surface-to-volume ratio might exhibit more side-effects, such as the generation of reactive oxygen species (ROS) [5–7]. Nevertheless, our current knowledge regarding the effects of nanoparticles on specific physical and mechanobiological aspects of the cell remains insufficient due to limitations in the available analytical methods.

Magnetic nanoparticles (MNPs) have been commonly used as biosensors as well as diagnostic and delivery tools in biomedicine and biotechnology [8–10]. More specifically, several biocompatible materials, such as silica, polyethyleneimine, and chitosan have been used to coat nanomaterials to both reduce side-effects and confer beneficial properties on bare nanomaterials [11–17].

Among MNPs, silica-coated MNPs containing rhodamine B isothiocyanate [MNPs@SiO_2_(RITC)], with layers of silica and a MNP core, are being used for separating and marking cells [13]. Based on traditional methods of evaluation of toxicity, such as the chromosome aberration test, haematoxylin and eosin staining, 3-(4,5-dimethylthiazol-2-yl)-2,5-diphenyltetrazolium bromide (MTT) test, and in vivo tissue distribution, MNPs@SiO_2_(RITC) has been reported to be nontoxic [18–22]. However, it was suggested that MNPs@SiO_2_(RITC) might exert biophysical side effects via the generation of ROS in cells [4, 21].

Briefly, ROS are known to oxidize membranous lipids as well as cytoskeleton proteins. In particular, ROS can oxidize polyunsaturated phospholipids, glycolipids, and cholesterol in the membrane [23], thereby reducing membrane fluidity and permeability [24, 25]. In addition, internalised nanoparticles have been shown to impair cytoskeleton proteins and interfere with focal adhesion kinase-mediated signalling [26]. With lamellipodia (branched actin filaments) and filopodia (extended finger-like protrusions), cell adhesion is known to occur due to the presence of focal adhesion (FA) complexes, with cell morphology being determined through the balance between adhesion and tension [27]. The involvement of adenosine triphosphate (ATP) in the movement of myosin over filamentous actin (F-actin) and in the polymerization of actin has suggested that cellular mechanics could be altered by ATP depletion [28]. In that regard, treatment with MNPs@SiO_2_(RITC) has been reported to have caused metabolic changes in cells, including ATP depletion [21].

The submicron elastomeric pillar array is considered an excellent tool for measuring cellular force as the nanometric level of pillar deflection can be calculated by incorporating optical microscope imaging [29, 30]. Furthermore, it can be used to analyse the initial contact of a cell with a substrate and has been reported to mimic continuous substrates of a specific rigidity [31]. Thus, the mechanobiological effects of nanoparticles on cells could be quantitatively studied by measuring the traction force using submicron elastomeric pillars [29, 31].

Assessment of potential side-effects of nanoparticles using classical methods is limited owing to delicate changes and complexities at the nano level. Thus, instead of focusing on targeted molecules, omics approaches, including genomics, transcriptomics, proteomics, and metabolomics, have been used in nanotoxicity studies [22]. However, restrictions in assessing the intricate signalling pathways and delicate events in cells and organisms still exist. For example, although transcriptomics can reflect enormous genotypic changes, it has been shown to be insufficient for understanding the actual phenotype [32, 33]. In contrast, in the case of metabolomics, which can constitute an endpoint feature of biological phenotypes [34, 35], no amplification methods are available for very low-abundance metabolites, and quantitative analysis of the targeted method might likely provide only a partial representation of the overall metabolism [36]. Accordingly, a combination of transcriptomics and metabolomics, termed "metabotranscriptomics", has been utilised for a more comprehensive analysis of cells and evaluation of nanotoxicity following treatment with nanoparticles [4, 21, 22].

We have previously analysed the effects of MNPs@SiO_2_(RITC) on HEK293 cells [4, 21, 37, 38]. In addition, HEK293 cells have also been extensively used in the study of nanoparticle-induced toxicity and in renal toxicity models [39, 40]. In the present study, we analysed membrane fluidity, which is an important indicator of biophysical changes, including cell freshness and cell deformability [41, 42], in MNPs@SiO_2_(RITC)-treated HEK293 cells using total internal reflection fluorescence microscopy (TIRFM). Moreover, we measured the changes in the traction force, which is known to directly reflect biophysical changes and has been highly correlated with changes in cell membrane in MNPs@SiO_2_(RITC)-treated HEK293 cells. Thus, metabotranscriptomics-based biophysical assessment was performed to comprehensively evaluate MNPs@SiO_2_(RITC)-induced effects on HEK293 cells.

## Results

### Characteristics of MNPs@SiO_2_(RITC) and silica NPs

Our generated MNPs@SiO_2_(RITC) nanoparticles consisted of an approximately 9-nm cobalt ferrite core (CoFe_2_O_3_) chemically bonded to rhodamine isothiocyanate dye (RITC) and coated with a silica shell [13]. Moreover, silica nanoparticles, which are identical to the silica shell of MNPs@SiO_2_(RITC), have been shown to exhibit similar biological effects to those exhibited by MNPs@SiO_2_(RITC) [4, 20, 21]. The diameter of both MNPs@SiO_2_(RITC) and silica nanoparticles was demonstrated to be 50 nm (Additional file 1: Fig. S1), with MNPs@SiO_2_(RITC) being reported to have a *zeta* potential of − 40 to − 30 mV [13, 20]. A previous study using inductively coupled plasma atomic emission spectrometry showed that approximately 10^5^ particles of MNPs@SiO_2_(RITC) per cell were internalised in MCF-7 breast cancer cells [13]. We determined the dosage used in the present study by treating HEK293 cells with MNPs@SiO_2_(RITC) at concentrations ranging from 0.01 to 2.0 µg/µL for 12 h and calculating their uptake efficiencies [21]. We accordingly found that the optimal concentration of MNPs@SiO_2_(RITC) for in vitro use was 0.1 µg/µL; this concentration had been previously used for MRI contrast without any reported toxicological effects in human cord blood-derived mesenchymal stem cells [43]. In addition, any disturbances in gene expression and metabolic profiles of treated cells at this concentration were similar to those observed in control cells [21]. We further observed that the uptake efficiency of MNPs@SiO_2_(RITC) plateaued at 1.0 µg/µL. Therefore, we used a low dose of 0.1 µg/µL and high dose of 1.0 µg/µL in the present study.

### MNPs@SiO_2_(RITC)-induced lipid peroxidation decreases cell membrane fluidity

We determined the optimal concentrations of MNPs@SiO_2_(RITC) for cell labelling, as well as the cellular uptake of nanoparticles during a 12 h-incubation to be 0.1 and 1.0 μg/µL, respectively, based on our previous studies using a fluorescence assessment method [4, 21]. We found that the viability of cells treated with 1.0 μg/µL MNPs@SiO_2_(RITC) was similar to that of cells with or without treatment with bare silica nanoparticles (NPs) of the same size (50 nm in diameter) (Additional file 1: Fig. S2), consistent with previous reports [4, 21].

**Fig. 1:**
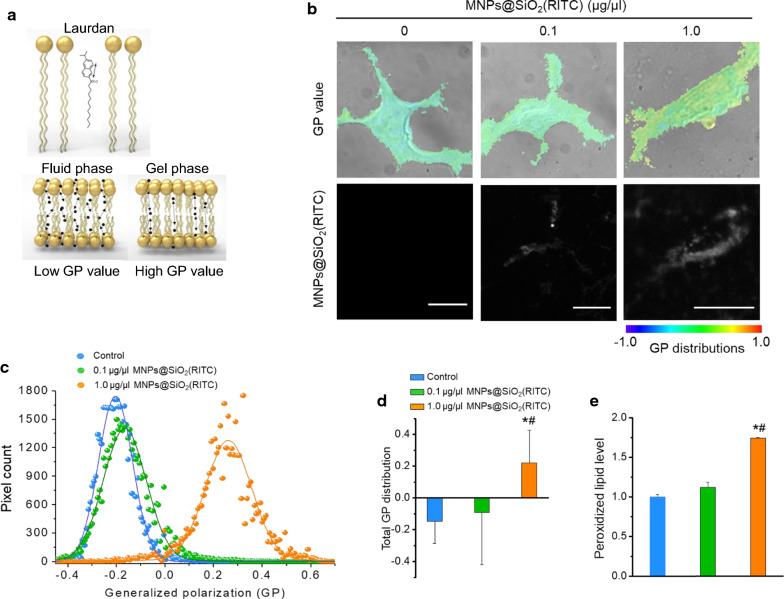
Laurdan GP images and GP frequency distributions of MNPs@SiO_2_(RITC)-treated HEK293 cells. **a** Schematic representation of laurdan usage for measuring the GP value of membranes. **b** Merged DIC and psuedo-coloured GP images (upper panel) of HEK293 cells. MNPs@SiO_2_(RITC) distributions are shown in each lower panel. GP distributions ranged from − 1.0 to 1.0. Scale bar = 2.5 µm. **c** GP frequency distributions of cells. GP values of each pixel are represented as dots and were fitted to Gaussian distributions. **d** Total GP values. Data represent the mean ± SD of 3 independent experiments (N = 10). **e** Evaluation of peroxidised lipids using ferrous thiocyanate. The intensity of ferrous thiocyanate alone was used as the blank. Data represent the mean ± SD of 3 independent experiments. *p < 0.05 *vs* untreated control, ^#^*p* < 0.05 compared between 0.1 and 1.0 µg/µL MNPs@SiO_2_(RITC)-treated cells

We analysed the changes in lipid peroxidation and membrane fluidity in treated HEK293 cells using total internal reflection fluorescence microscopy (TIRFM). We furthermore investigated the changes in cell membrane fluidity after treatment with MNPs@SiO_2_(RITC) by measuring the values of 6-dodecanoyl-2-dimethylaminonaphthalene (laurdan) generalised polarization (GP) using TIRFM (Fig. 1a). We accordingly noted that the number of high-GP areas on the cell surface, corresponding to rigid domains, increased on treatment with MNPs@SiO_2_(RITC); in particular, abundantly distributed regions of MNPs@SiO_2_(RITC) were found to be primarily colocalised with high GP-distributed regions at a GP scale of −1.0 to 1.0 (Fig. 1b, Additional file 1: Fig. S3). We subtracted the GP frequency distribution values of treated cells from the corresponding values of untreated control cells to obtain frequency difference curves (Fig. 1c) and total mean GP values (Fig. 1d). We observed a similar trend in the relative levels of peroxidised lipids (Fig. 1e).

A previous study revealed that MNPs@SiO_2_(RITC) induced the generation of intracellular ROS via mitochondria dysfunction in HEK293 cells. In particular, generation of ROS in cells treated for 12 h was attributed to the shell of MNPs@SiO_2_(RITC) and silica nanoparticles (silica NPs) rather than the cobalt ferrite core [4, 21]. Thus, to determine the MNPs@SiO_2_(RITC) treatment-induced generation of intracellular ROS, we performed 2′,7′-dichlorodihydrofluorescein diacetate staining in HEK293 cells treated with MNPs@SiO_2_(RITC) and silica NPs. We observed that the level of intracellular ROS was increased by over 50% upon treatment with both 1.0 µg/µL MNPs@SiO_2_(RITC) and silica NPs compared with that in untreated control and 0.1 µg/µL MNPs@SiO_2_(RITC)- and silica NPs-treated cells (Additional file 1: Fig. S4). In addition, we did not observe any significant difference in the treatment with 1.0 µg/µL MNPs@SiO_2_(RITC) and silica NPs, in line with previous reports [4, 21]. These results indicated that the rigid regions in the plasma membrane were increased through lipid peroxidation induced by ROS generated from the shell of MNPs@SiO_2_(RITC).

### Treatment with MNPs@SiO_2_(RITC) decreased cell aspect ratio and spread area but increased traction force

**Fig. 2:**
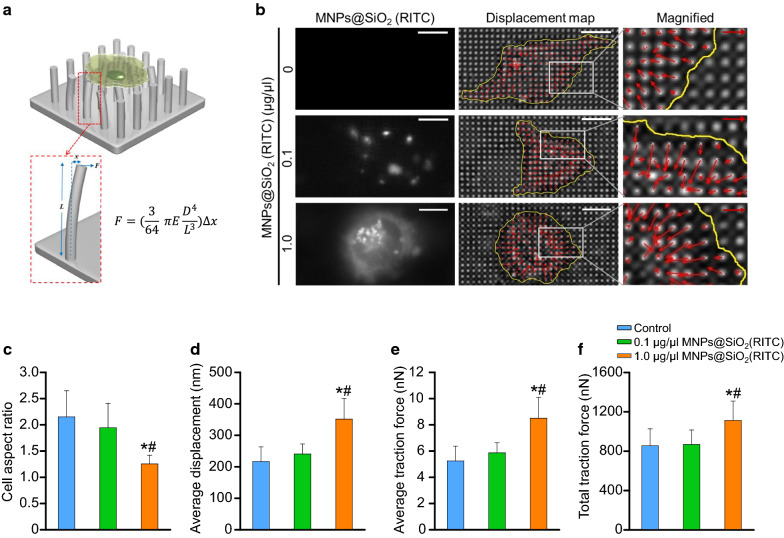
Change in pillar deflection, traction force, aspect ratio, and surface area of MNPs@SiO_2_(RITC)-treated HEK293 cells. **a** Schematic drawing of the measurement of the traction force (*F*) of cells treated with MNPs@SiO_2_(RITC) using submicron elastomeric micropillars. **b** Representative images of the concentration of MNPs@SiO_2_(RITC) inside the cell, pillar deflections, and magnified pillar deflections at the edge of the cell (left to right). Red arrows represent 356 nm of pillar deflection, whereas the white bar represents 8 µm. Yellow lines indicate the approximate cell boundary. The direction and length of the red arrow indicate the magnitude and direction of pillar deflection, respectively. **c** Aspect ratio of cells spread over the pillar array. **d** Average displacement of each pillar under the cell. **e** Average traction force of each pillar under the cell and **f** total traction force of pillars beneath the cell. Data represent the mean ± SD. *p < 0.05 vs untreated control. ^#^*p* < 0.05 compared between 0.1 and 1.0 µg/µL MNPs@SiO_2_(RITC)-treated cells

As membrane fluidity is known to be closely related to cell morphology and focal adhesion [44, 45], we investigated whether a decrease in MNPs@SiO_2_(RITC)-mediated membrane fluidity would also affect cell morphology and focal adhesion. We evaluated the effects of MNPs@SiO_2_(RITC) on cell polarity as local cell contraction is tightly associated with these activities along with changes in focal adhesion [46]. Moreover, we measured the cell aspect ratio. We analysed images of cells and submicron pillars at 12 h after cell seeding (Fig. 2a, b). We found that the aspect ratio of cells treated with 0.1 µg/µL MNPs@SiO_2_(RITC) did not significantly differ from that of untreated control cells. However, the ratio of cells treated with 1.0 µg/µL MNPs@SiO_2_(RITC) was shown to be significantly smaller than that of untreated control cells (Fig. 2c).

We then used pillar deflection in the magnified images to measure pillar displacement (Fig. 2d) and calculate traction force (Fig. 2e, f). To calculate pillar traction force, the displacement of each pillar was multiplied with the pillar bending stiffness [31]. In particular, we noted that 1.0 µg/µL MNPs@SiO_2_(RITC)-treated cells showed an increase in pillar displacement (351 ± 65 nm; mean ± SD), which was significantly higher than that in untreated control cells (216 ± 46 nm). The average (8.5 ± 2 nN) and total (1112 ± 197 nN) traction forces in 1.0 µg/µL MNPs@SiO_2_(RITC)-treated cells were significantly higher than those (5.2 ± 1 and 855 ± 172 nN) in untreated control cells, indicating that cell traction force was affected by 1.0 µg/µL MNPs@SiO_2_(RITC). Taken together, the increase in cell traction force was suggested to have been caused by the MNPs@SiO_2_(RITC) treatment-induced reduction in cell polarity and spread area.

### Treatment with MNPs@SiO_2_(RITC)- impaired cell movement

**Fig. 3:**
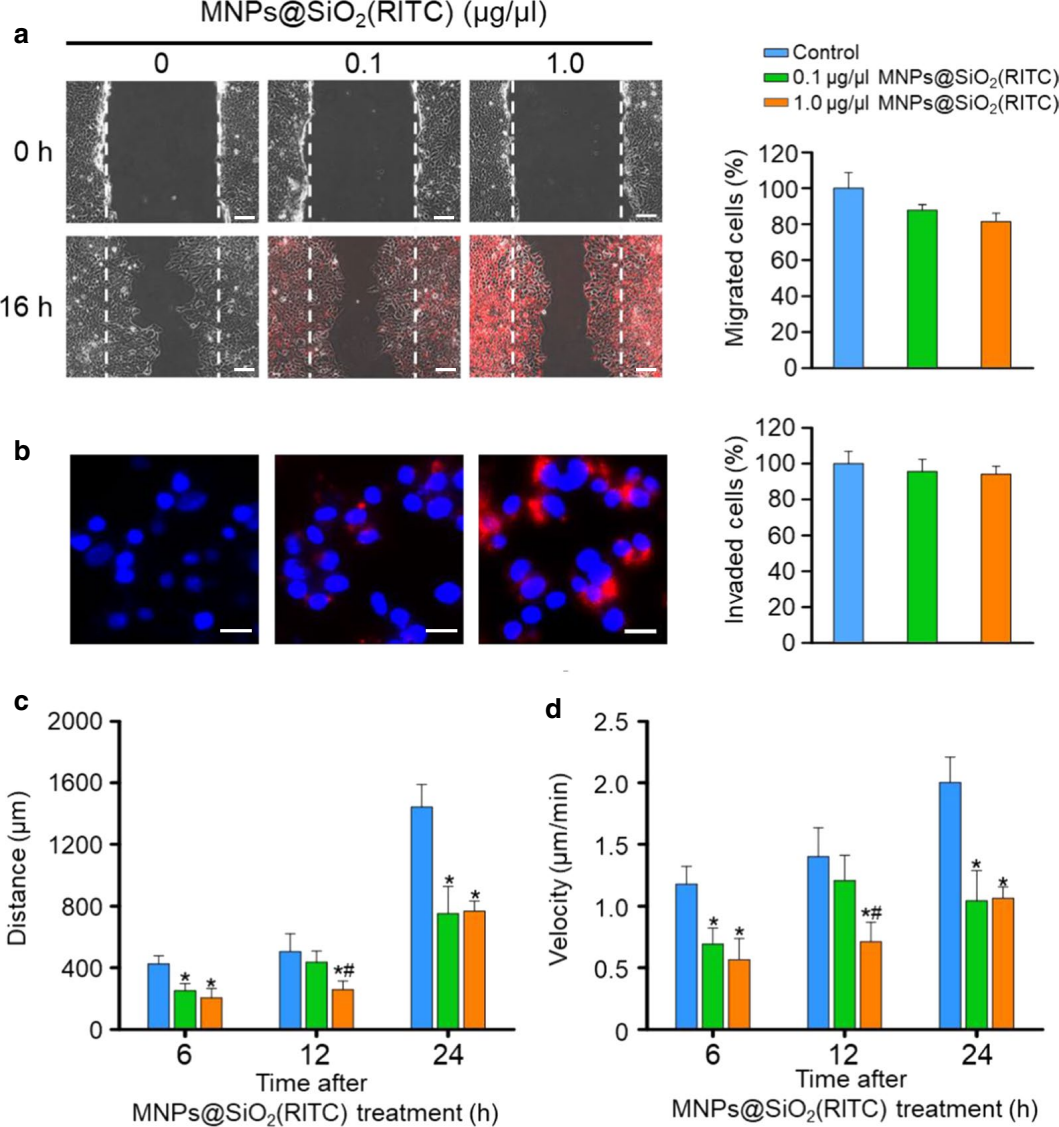
Evaluation of the movement of MNPs@SiO_2_(RITC)-treated HEK293 cells. **a** Representative images of the scratch assay and quantitative image analysis. Images of the initial scratched (0 h) layer are shown in the upper panels. Images of HEK293 cells after treatment with MNPs@SiO_2_(RITC) for 16 h are shown in the lower panels. Scale bar = 100 μm. Quantitative image analysis of migrated cells in MNPs@SiO_2_(RITC)-treated HEK293 cells is shown in the bar graph. **b** Representative images of HEK293 cells and quantitative image analysis of the invasion assay results after treatment with MNPs@SiO_2_(RITC) for 12 h. Red, MNPs@SiO_2_(RITC); blue, Hoechst 33,342. Scale bar = 20 μm. Quantitative image analysis of invaded MNPs@SiO_2_(RITC)-treated HEK293 cells is shown in the bar graph. Individual cell tracking analysis for travel distance (**c**) and velocity (**d**) of MNPs@SiO_2_(RITC)-treated HEK293 cells at 6, 12, and 24 h. Data represent mean ± SD of 3 independent experiments. *p < 0.05 *vs*. untreated control, #*p* < 0.05 compared between 0.1 and 1.0 µg/µL MNPs@SiO_2_(RITC)-treated cells

We previously analysed the effect of treatment with MNPs@SiO_2_(RITC) on the migratory activity of human bone marrow-derived mesenchymal stem cells (hBM-MSCs). Using conventional assays, such as scratch and invasion assays, we observed an impairment in the migratory activity of hBM-MSCs [42]. To evaluate the biophysical changes related to biological functions of HEK293, we analysed the effect of 0.1 or 1.0 µg/µL MNPs@SiO_2_(RITC) on the movement of HEK293 cells using conventional assays. Accordingly, using the scratch assay, we did not observe any difference in the migratory activity between MNPs@SiO_2_(RITC)-treated HEK293 cells and untreated control cells (Fig. 3a). Similarly, no difference was observed between the invasion ability of MNPs@SiO_2_(RITC)-treated HEK293 cells and untreated control cells analysed using the transwell invasion assay (Fig. 3b).

However, it should be mentioned that the aforementioned results of the scratch and invasion assays did not exclude the cell growth effect and treatment with growth-arrest agents, such as mitomycin C, as these assays are known to be highly toxic to HEK293 cells [47]. Thus, we analysed the movement of individual cells by tracking cells on pillars for 24 h after treatment with MNPs@SiO_2_(RITC) for 12 h on a dish (Additional files 2, 3, 4: Movie S1, Movie S2, and Movie S3). We remarked that the distances travelled by cells were decreased to a significantly greater extent in MNPs@SiO_2_(RITC)-treated HEK293 cells compared with untreated control cells at both 6 and 24 h (Fig. 3c). In addition, we obtained similar results for movement speeds at 6 h and 24 h (Fig. 3d).

### Treatment with MNPs@SiO_2_(RITC) decreased intracellular level of ATP

**Fig. 4:**
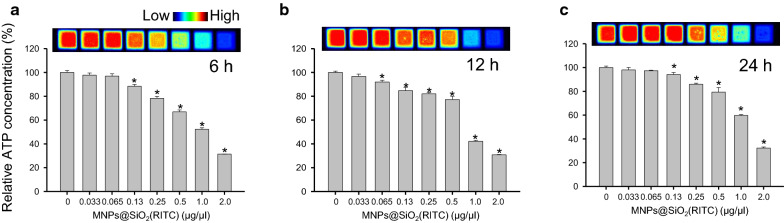
Evaluation of intracellular level of ATP in MNPs@SiO_2_(RITC)-treated HEK293 cells. HEK293 cells were treated with 0 to 2.0 μg/µL MNPs@SiO_2_(RITC) for **a** 6, **b** 12, and **c** 24 h, and the intracellular ATP level was evaluated. Luminance was captured and expressed as a pseudo-colour scale image. Data represent mean ± SD of 3 independent experiments. *p < 0.05 *vs*. untreated control, ^#^*p* < 0.05 compared between 0.1 and 1.0 µg/µL MNPs@SiO_2_(RITC) -treated cells

To evaluate the changes in the intracellular level of ATP in MNPs@SiO_2_(RITC)-treated cells, HEK293 cells were treated with MNPs@SiO_2_(RITC) at concentrations ranging from 0 to 2.0 µg/µL for 6, 12, and 24 h (Fig. 4). We found that intracellular levels of ATP were decreased in a dose-dependent manner in MNPs@SiO_2_(RITC)-treated cells, starting from the 0.13 µg/µL dose. Moreover, we observed that the decrement pattern was similar for 6, 12, and 24 h treatments.

### Metabotranscriptomic network of MNPs@SiO_2_(RITC)-treated HEK293 cells

**Fig. 5:**
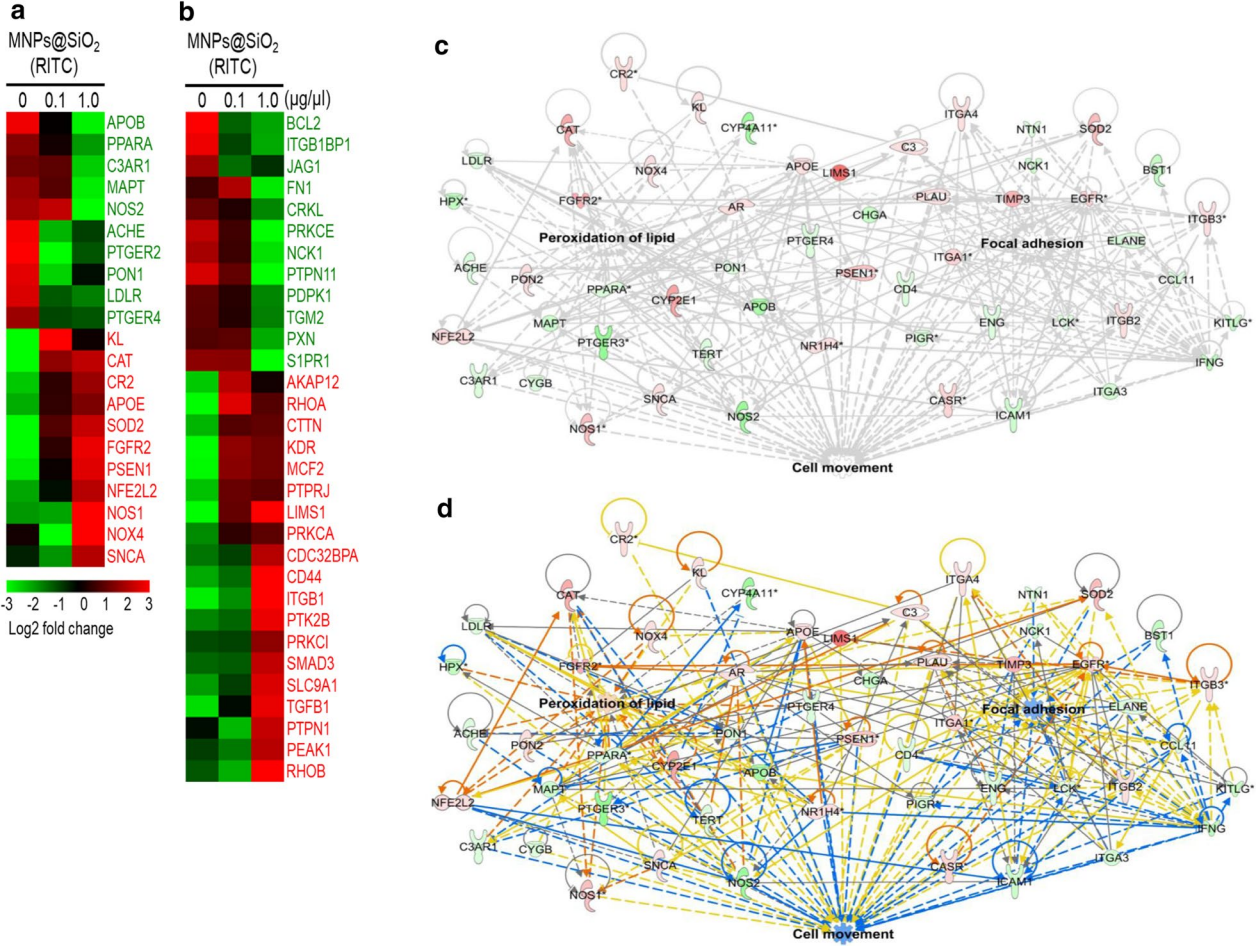
Transcriptome analysis in HEK293 cells treated with MNPs@SiO_2_(RITC) for 12 h. Heatmap of differentially expressed genes (21 genes related to lipid peroxidation (**a**) and 31 genes related to focal adhesion (**b**)) at 0.1 and 1.0 µg/µL MNPs@SiO_2_(RITC)-treated cells, according to microarray analysis. Red and green areas indicate up- and downregulated genes, respectively. A network of lipid peroxidation and focal adhesion-related genes was constructed algorithmically using IPA. **c** The transcriptome network of 1.0 µg/µL MNPs@SiO_2_(RITC)-treated cells and **d** prediction analysis for the network are shown. Red and green areas indicate up- and downregulated genes, respectively. Orange and blue colours indicate activation and suppression, respectively. Lines indicate indirect (dotted) or direct (solid) relationship. Differentially expressed genes obtained from microarray data (genes with > threefold change) are shown

To analyse the dispersion phenomena of MNPs@SiO_2_(RITC)-treated cells, we constructed a coexpression network of genes and metabolites using a transcriptome generated from microarray analysis and the metabolome derived from amino acid and organic acid profiling through the Ingenuity Pathway Analysis (IPA, http://www.ingenuity.com) [21]. Microarray expression analysis showed that the levels of 21 and 31 genes associated with lipid peroxidation and focal adhesion formation, respectively, were altered in MNPs@SiO_2_(RITC)-treated cells (Fig. 5a, b). Using a threefold cut-off only to determine changes in the transcriptomic network, we detected more pronounced changes in 1.0 µg/µL MNPs@SiO_2_(RITC)-treated cells compared with 0.1 µg/µL MNPs@SiO_2_(RITC)-treated and control cells; these changes were related to cell movement (Fig. 5c, Additional file 1: Fig. S5). In silico prediction of the network revealed activation of lipid peroxidation, as well as suppression of focal adhesion and cell movement in 1.0 µg/µL MNPs@SiO_2_(RITC)-treated HEK293 cells (Fig. 5d).

**Fig. 6:**
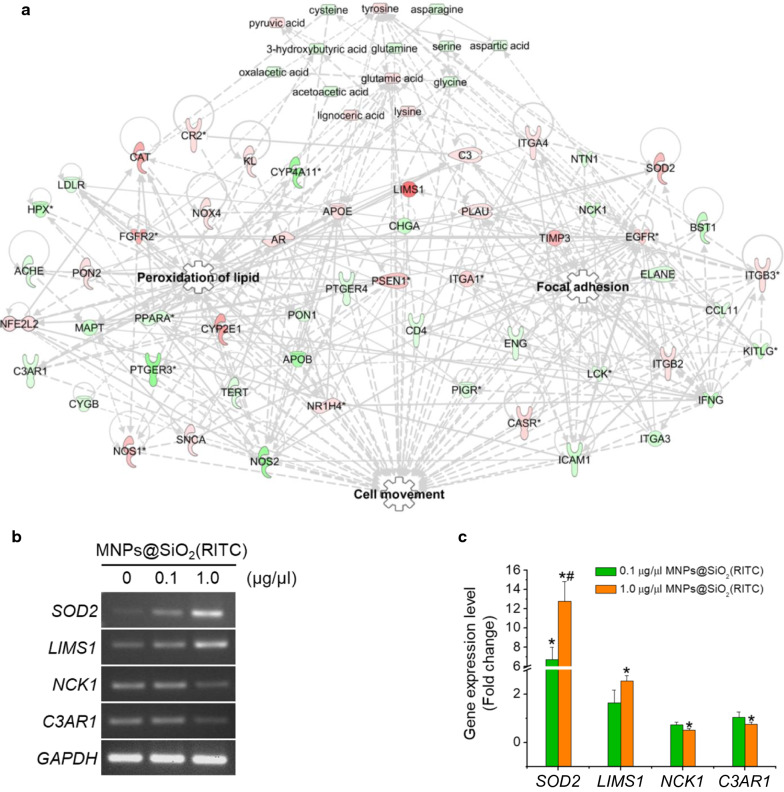
Metabotranscriptomic analysis of the microarray and metabolite profile of MNPs@SiO_2_(RITC)-treated cells. **a** Lipid peroxidation and focal adhesion-related genes and metabolite networks were algorithmically constructed using IPA. Red and green areas indicate up- and downregulated genes, respectively. Lines indicate indirect (dotted) or direct (solid) relationship. Differentially expressed genes obtained from microarray data (> threefold change) and disturbances in the metabolic profile (> 20% change) are shown. Quantitative evaluation of the metabotranscriptomic network-related genes by **b** semi-quantitative reverse transcription (RT)-PCR and **c** quantitative real-time PCR. Cells were treated with 0.1 and 1.0 μg/µL MNPs@SiO_2_(RITC) for 12 h. RT-PCR and qPCR were performed using gene-specific primer pairs for *SOD2*, *LIMS1*, *NCK1*, and *C3AR1*. *GAPDH* was used as the internal control. PCR products were normalised relative to the levels of internal control. Data represent the mean ± SD of 3 independent experiments. *p < 0.05 vs untreated control. Transcriptome and metabolome data are reproduced from our previous study, Copyright © 2019 *Springer Nature* [37]

Although transcriptomics provided comprehensive information regarding MNPs@SiO_2_(RITC)-treated cells, the data obtained were only qualitative. Thus, for a network-based evaluation, we used a combination of transcriptome and metabolome networks, termed the metabotranscriptomic network, through substitution of amino acid and organic acid profiles (cut-off ± 20% change), as described in our previous report [21]. In the group treated with 1.0 µg/µL MNPs@SiO_2_(RITC), we found that the amino acid, organic acid, and free fatty acid profiles showed increased levels of tyrosine, pyruvic acid, glutamic acid, lysine, and lignoceric acid; in contrast, we observed decreased levels of cysteine, asparagine, 3-hydroxybutyric acid, glutamine, serine, aspartic acid, oxaloacetic acid, glycine, and acetoacetic acid [37]. This combined network provided more reliable information than the transcriptomic network alone, allowing also the detection of more pronounced changes in 1.0 µg/µL relative to 0.1 µg/µL MNPs@SiO_2_(RITC)-treated and control cells (Fig. 6a, Additional file 1: Fig. S6, Table S1). In silico prediction of the integrated network also revealed a similar trend to that observed in the transcriptome network (Additional file 1: Fig. S7).

We subsequently quantified the expression level of genes associated with the network using semi-quantitative reverse transcription PCR (Fig. 6b) and quantitative (q) PCR (Fig. 6c). In particular, we found that expression levels of superoxide dismutase 2 (*SOD2*) and LIM zinc finger domain-containing 1 (*LIMS1*) were increased, whereas those of NCK adaptor protein 1 (*NCK1*) and complement C3a receptor 1 (*C3AR1*) were decreased in the 1.0 µg/µL MNPs@SiO_2_(RITC)-treated cells relative to those in untreated control cells.

## Discussion

The present study demonstrated the effects of nanoparticles on the biophysical properties of cells. In addition, functional analysis using the metabotranscriptomics approach allowed us to deduce inter-connective relationships between genes and metabolites associated with lipid peroxidation, focal adhesion, and cell movement in MNPs@SiO_2_(RITC)-treated cells.

We observed that the 1.0 µg/µL MNPs@SiO_2_(RITC)-treated cells exhibited markedly increased levels of lipid peroxidation compared with the 0.1 µg/µL MNPs@SiO_2_(RITC)-treated cells and untreated control. These results indicated that exposure of cells to 1.0 µg/µL MNPs@SiO_2_(RITC) might impair certain biological functions due to the production of ROS and lipid peroxidation. This was consistent with the finding that oxidative stress induces lipid peroxidation, which is often observed in disease states, such as aging, sickle-cell disease, malaria, and diabetes [48–50]. It was also consistent with the decrease in the deformability of hBM-MSCs over time owing to the generation of intracellular ROS and lipid peroxidation, which was shown to lead to decreased membrane fluidity and deteriorated cell quality [41]. Moreover, we previously showed that ROS-distributed regions distinctly co-localised with MNPs@SiO_2_(RITC)-distributed regions in cells [21]; hence, we assumed that regions of abundantly distributed MNPs@SiO_2_(RITC) might be primarily co-localised with high GP-distributed regions along with lipid peroxidation, thus distinguishing damaged membrane regions from intracellular regions. Thus, our results suggested that exposure of cells to 1.0 µg/µL MNPs@SiO_2_(RITC) could potentially impair the biological functions of exposed cells by increasing their membrane rigidity.

Nanoparticle-induced cell membrane damage is reportedly caused through direct interactions between membrane lipids and nanoparticles [51]. Further, silica and gold nanoparticles are known to penetrate cell membranes [52, 53]. In particular, 50-nm NPs were demonstrated to be likely internalised through endocytic pathways as nanoparticles > 10 nm were found to be coated directly with the plasma membrane and be internalized via clathrin- and caveolae-mediated endocytic pathways [54, 55]. In this study, the altered gene expression of the clathrin-mediated endocytic pathway was reported to significantly alter the internalization of MNPs@SiO_2_(RITC)s in HEK293 cells and SH-SY5Y neuroblastoma cells [4]. Thus, we proposed that MNPs@SiO_2_(RITC) enter cells through endocytosis; this might constitute one reason for the observed decreased membrane fluidity because membrane tension regulates membrane deformation or changes in cell shape (e.g., endocytosis, exocytosis, cytokinesis, and cell motility) [56].

As the membrane and cytoskeleton are known to be tightly associated with phosphoinositides, the status of cell membranes has been highly linked to the regulation of the cytoskeleton [57, 58]. Nanoparticle-induced lipid peroxidation has been reported to alter cell morphology and membrane roughness in human lymphocytes [59]. We accordingly found that the cell aspect ratio and cell spread area were decreased in MNPs@SiO_2_(RITC)-treated cells, which exhibited a round morphology, suggesting that focal adhesion was impaired owing to the imbalance between adhesion and tension caused by treatment with MNPs@SiO_2_(RITC) treatment.

Nanoparticles have also been shown to increase traction force but inhibit cellular migration [60]. Traction force is known to be primarily regulated by phosphorylation of myosin II through the assembly (dephosphorylated myosin) and disassembly (phosphorylated myosin) of the myosin light chain (also termed the myosin head) and actin filaments, with these processes obtaining energy from the hydrolysis of ATP [61, 62]. Our previous study showed that the intracellular level of ATP in 1.0 µg/µL MNPs@SiO_2_(RITC)-treated cells was decreased to approximately 50% due to mitochondrial damage [21]. In the present study, we observed that treatment with MNPs@SiO_2_(RITC) decreased the intracellular levels of ATP in dose- and time-dependent manners. This might have been one of the factors responsible for the observed increment in the total traction force and reduction in cell movement activity.

There were limitations in interpreting the actual phenotype obtained in the transcriptome network associated with lipid peroxidation, focal adhesion, and cell movement. Thus, we combined datasets providing information on the interactions between differentially expressed genes and altered metabolites, and generated a metabotranscriptomic network. In particular, the network showed that the expression levels of *SOD2*, which is tightly associated with oxidative stress [63], and *LIMS1*, which is an important molecule in the linkage between actin and integrin for the formation of focal adhesion [64], were increased, whereas the levels of *NCK1*, an upstream regulator in the formation of actin-rich protrusions in the plasma membrane [65], and *C3AR1*, which is reduced in oxidative conditions [66, 67], were decreased in 1.0 µg/µL MNPs@SiO_2_(RITC)-treated cells relative to those in untreated controls. Thus, the convergence of the metabolic profiles and transcriptome disturbances allowed interpretation of the changes in the movement of MNPs@SiO_2_(RITC)-treated cells.

Dose justification is determined based on uptake (labelling) efficiency, and using a fluorescence assessment method, we previously reported that the plateau of uptake (labelling) occurred at 1.0 μg/μL MNPs@SiO_2_(RITC)-treated HEK293 cells [21]. Moreover, with respect to toxicity, reduction in the cell viability of MNPs@SiO_2_(RITC) treated HEK293 cells was not identified. However, changes in ATP levels were observed with respect to NP concentration, that is, an approximately 50% decrement in the level of ATP in 1.0 μg/μL MNPs@SiO_2_(RITC)-treated HEK293 cells exhibiting a plateau uptake, whereas an approximately 5% decrement in the level of ATP in 0.1 μg/μL MNPs@SiO_2_(RITC)-treated HEK293 cells with an adequate uptake.

The effects of MNPs@SiO_2_(RITC) and silica NPs on HEK293 cells were similar with respect to cell viability and ROS generation. In addition, cellular localization of MNPs@SiO_2_(RITC) and silica NPs indicated that both types of NPs were accumulated in lysosomes (Additional file 1: Fig. S8). Consistent with our previous reports, we postulated that the effects of MNPs@SiO_2_(RITC) were exerted by silica, which is a peripheral part of the MNPs@SiO_2_(RITC) [4, 21, 42].

The decrease in moving speed was recovered in control and MNPs@SiO_2_(RITC)-treated HEK293 cells in a time-dependent manner. This could be explained by a couple of possibilities. One possibility is that cells adapted to the altered conditions. Another possibility is that the MNPs@SiO_2_(RITC)-induced decrement in moving speed changed phases in a time-dependent manner [6 h (acute phase), 12 h (intermediate phase), and 24 h (chronic phase)]. As shown in the changes in the concentration of ATP (Fig. 4), the decreased levels of ATP were recovered, but not fully. These possibilities might have been reflected in the moving speed of MNPs@SiO_2_(RITC)-treated HEK293 cells.

The possibility of the dye and cobalt ferrite leaching out of MNPs@SiO_2_(RITC) and exerting toxic effects in cells exposed to these nanoparticles for a long time has been a main concern. For instance, the dye signal of MNPs@SiO_2_(RITC) was reduced by approximately 90 and 40% in human cord blood-derived mesenchymal stem cells during their third and seventh passages, respectively. These observed reductions might have been due to the dilution effect generated by cell proliferation [19]. As there have been reports that treatment with MNPs@SiO_2_(RITC) did not cause any significant loss and measurable toxicological effects in HEK293 cells for 7 d and in a mouse model for 4 wk, respectively, it was assumed that the highly toxic cobalt ferrite substance did not leach out of the core of MNPs@SiO_2_(RITC) [4, 18, 21]. Based on these reports, we assumed that our MNPs@SiO_2_(RITC) nanoparticles were also stable enough to allow the discernment of subtle toxicity in cells exposed to MNPs@SiO_2_(RITC) for a long time.

## Conclusions

In conclusion, our results demonstrated a reduction in membrane fluidity, abnormal focal adhesion, and decrement in cell movement upon treatment with a high (1.0 µg/µL) concentration of MNPs@SiO_2_(RITC) nanoparticles. Our findings indicated that nanoparticles should be used at the lowest possible dosage in therapeutic or diagnostic applications to prevent potential nanotoxicity. Our comprehensive approach of toxicological evaluation will aid the future assessment of nanoparticle sensitivity and associated potential toxicity. Moreover, our findings on nanotoxicity will be beneficial in terms of the development of safe nanoparticles for biomedical applications.

## Materials and methods

### Measurement of membrane fluidity

Changes in membrane fluidity were measured using laurdan, a fluorescent dye that exhibits a 60-nm spectral shift from disordered to ordered bilayer phases, and an in-house combined differential interference contrast-total internal reflection fluorescence microscopy experimental system (DIC-TIRFM) [41, 68]. The procedure was based on a well-described protocol [41, 69, 70]. Briefly, cells were seeded on cover slips (no. 1 thickness, 0.13–0.16 mm) and treated with 0.1 and 1.0 μg/µL MNPs@SiO_2_(RITC) for 12 h. For staining with laurdan, cells were incubated with medium containing 10 µM laurdan at 37 °C and 5% CO_2_ for 2 h. Cells were washed twice with phosphate buffered saline (PBS) and fixed with fixation buffer (Cytofix; BD, San Jose, CA, USA). Cover slips containing cells were mounted onto other cover slips (no. 1 thickness, 0.13–0.16 mm) using mounting medium (Prolong gold antifade; Molecular Probes, Eugene, OR, USA). Cell morphological changes, laurdan fluorescence, and MNPs@SiO_2_(RITC) distribution were observed using an oil-type 100 × objective lens (Olympus UPLFL 100 × /1.3 N.A., W.D. 0.1 mm, Tokyo, Japan) and a CCD camera (QuantEM 512SC, Photometrics, Tucson, AZ, USA). Laurdan was excited at 405 nm, and emission fluorescence was detected with 420 nm and 473 nm bandpass filters (resolution: ± 5 nm). As a parameter of membrane fluidity, GP [(Intensity_420 nm_ − Intensity_473 nm_)/(Intensity_420 nm_ + Intensity_473 nm_)] was calculated and pseudo-coloured; GP images merged with DIC images were generated using the Image J software (NIH, Bethesda, MD, USA) [71]. Gaussian distributions were generated using the nonlinear fitting algorithm in Sigma plot 10.0 (Systat Software Inc., San Jose, CA, USA).

### Microfabrication of submicron elastomeric pillar array

Photolithography was used to fabricate a silicon wafer mould with a series of holes [72]. To make the pillars, polydimethylsiloxane (PDMS) was mixed in a 10:1 ratio with its curing agent (Sylgard 184; Dow Corning, Midland, MI, USA) and vacuumed for 15 min; the mould was then spin-coated with PDMS at 1,000 RPM for 1 min and again vacuumed for 30 min to remove bubbles. The mould with PDMS was cured at 80 °C for 3 h. The pillar array was manufactured using this method. Each pillar was 900 nm in diameter (*D*), 1 µm in height (*L*), and 1.8 µm in centre-to-centre distance. To calculate the bending stiffness (*k*) of the pillar, the Euler–Bernoulli beam theory was applied [31]:

$$k = \frac{3}{64}{ }\pi E\frac{{D^{4} }}{{L^{3} }}$$

where *E* is the Young’s modulus (2 MPa) of the cured PDMS. Using this equation, the *k* of the pillar was calculated to be 24.2 nN/µm.

### Measurement of cell aspect ratio

The cell aspect ratio was measured by representing the area of the entire cell with a comparable elliptical shape using ImageJ (NIH); the division from the major axis of the ellipse to the minor axis was then taken as the cell aspect ratio.

### Measurement of traction force

Pillar images were acquired using a fluorescence microscope (Deltavision, GE Healthcare, Chicago, IL, USA) and a camera (CoolSNAP HQ^2^, Photometrics) at 37 °C and 5% humidity with a live cell chamber at 1 Hz. The location of each pillar in each frame was determined using the pillar tracking plugin (PillarTracker 1.1.3 version) for ImageJ. In the pillar reconstruction algorithm, the PillarTracker works to establish the exact grid of the pillar array, thereby allowing users to automatically detect and track the pillar locations. Throughout this study, pillars that had no contact with cells were used as reference pillars. To account for stage drift, the average displacement of reference pillars was deducted from the displacement data of pillars deflected by cells. To avoid the unwanted displacement of pillars by MNPs@SiO_2_(RITC), MNPs@SiO_2_(RITC)-treated cells were washed five times using PBS (Sigma-Aldrich, St. Louis, MO, USA) before seeding onto the pillar array. The displacement of each pillar was multiplied with its bending stiffness to calculate the traction force (*F*).

### Scratch assay

HEK293 cells were seeded and cultured to 100% confluence in 6-well plates. Cell monolayers were scratched using 200-μL micropipette tips. Media were replaced with serum-free media, and HEK293 cells were treated with MNPs@SiO_2_(RITC) for 16 h. Images of scratched areas were captured both before treatment (0 h) and after treatment (16 h) using an Axio Vert 200 M fluorescence microscope (Zeiss, Jena, Germany). Migration activity was quantified by counting the number of migrated cells from the initial scratch.

### Transwell invasion assay

Cell invasion activity was analysed using an 8-µm pore size Transwell polycarbonate membrane (Corning, CA, USA), as in a previous study [73]. The upper side of the insert was coated with Matrigel (1:10 dilution in 0.01 M Tris pH 8.0, 0.7% NaCl) for 2 h at 37 °C. Subsequently, 1 × 10^3^ HEK293 cells were treated with MNPs@SiO_2_(RITC) in serum-free medium for 12 h and seeded onto the insert. Then, 10% FBS-containing medium was added to the lower chamber as a chemoattractant. Cells were incubated for 12 h at 37 °C to allow invasion. The upper side of the membrane was washed with a cotton swab, and invaded cells on the lower side of the membrane were fixed in Cytofix buffer (BD, San Jose, CA, USA) and stained with 10 μg/μL Hoechst 33342 for 15 min at 25 °C. Images were acquired using an Axio Vert 200 M fluorescence microscope (Zeiss, Jena, Germany). The number of invaded cells was counted using the ImageJ software (NIH).

### Cell tracking

PDMS pillars were coated with fibronectin for 1 h before seeding of cells. Cells were seeded at a density of 3000 cells per sample and tracked every 5 min for 6 h on a JuLi™ Br live cell movie analyser (NanoEntek, Inc., Seoul, Korea). Finally, cell movement speed was calculated by tracking cells using the ImageJ software (NIH).

### Metabotranscriptomic data analysis

Differences in the gene expression of cells were examined using the Affymetrix system (ISTECH, South Korea) in conjunction with the Human U133 Plus 2.0 50 K microarray, which contains 54,675 probes. Differences in the data distribution were analysed using the GenPlex 3.0 software [21], with the probe signals being quantile-normalised. Amino acid and organic acid profiles were imported from previously reported data [21]. Biological pathways and functions were identified using the Ingenuity pathway analysis web-based bioinformatics software (IPA; Ingenuity Systems, Redwood City, CA, USA). Three-fold change in gene expression and 20% change in metabolites were used as cut-offs to generate datasets of significantly altered genes and metabolites.

### Statistical analysis and error correction

Results were analysed by one-way analysis of variance (ANOVA) with Bonferroni's multiple-comparison test using the IBM-SPSS software (IBM Corp., Armonk, NY, USA). *p* < 0.05 was considered statistically significant. In the experiments using micropillars, errors in the pillar deflections were corrected by reducing the average pillar deflection of pillars outside the cell.

## Supplementary Information

**Additional file 1: Table S1.** Ingenuity Pathway Analysis-based profiles of transcriptomic network-related genes in MNPs@SiO_2_(RITC)-treated cells. **Table S2.** RT-PCR and real-time PCR primer sequences for genes encoding transcriptomic network-related genes. **Figure S1.** Determination of size and homogeneity for MNPs@SiO_2_(RITC) and silica NPs using transmission electron microscope (TEM) analysis. TEM images of MNPs@SiO_2_(RITC) (a) and silica NPs (b). Scale bar = 50 nm. **Figure S2.** Evaluation of cytotoxicity in silica NPs and MNPs@SiO_2_(RITC) treated HEK293 cells. HEK293 cells were treated with silica NPs and MNPs@SiO_2_(RITC) for 12 h. The changes in cell viability were evaluated with MTS. Data represent mean ± SD of three independent experiments. N.S: Not significant. **Figure S3.** Low magnification laurdan GP images. Merged DIC and TIRFM images (upper panel) of HEK293 cells. Distributions of MNPs@SiO_2_(RITC) are shown in each lower panel. GP distributions ranged from −1.0 to 1.0. Scale bar= 2.5 µm. **Figure S4.** ROS generation in silica NPs and MNPs@SiO_2_(RITC)-treated cells. Evaluation of intracellular ROS generation using DCFH-DA after 12 h silica NPs and MNPs@SiO_2_(RITC) treatment in HEK293 cells. The intensity of non-oxidised DCFH-DA was used as a blank. Data represent mean ± SD of three independent experiments. *p < 0.05 vs non-treated control, ^#^p < 0.05 compared between 0.1 and 1.0 µg/µl of NPs-treated cells. N.S: Not significant. **Figure S5.** Transcriptomic analysis of microarray in HEK293 cells treated with MNPs@SiO_2_(RITC) for 12 h. Network of lipid peroxidation and focal adhesion related genes was constructed algorithmically by IPA. (a) Transcriptome network of 0.1 µg/µl MNPs@SiO_2_(RITC)-treated cells and (b) prediction analysis for the network. Red and green areas indicate up- and downregulated genes, respectively. Orange and blue colours indicate activation and suppression. The lines indicate indirect (dotted) or direct (solid) relationship. Differentially expressed genes obtained from microarray data (genes with > 3-fold change) are shown. **Figure S6.** Metabotranscriptomic analysis of microarray and metabolite profiles in cells treated with 0.1 µg/µl MNPs@SiO_2_(RITC) for 12 h. (a) Lipid peroxidation and focal adhesion related genes and metabolites network were constructed algorithmically by IPA in 0.1 µg/µl MNPs@SiO_2_(RITC)-treated HEK293 cells and (b) prediction analysis for the network. Red and green areas indicate up- and downregulated genes, respectively. Orange and blue colours indicate activation and suppression, respectively. The lines indicate indirect (dotted) or direct (solid) relationship. Differentially expressed genes obtained from microarray data (> 3-fold change) and disturbances in metabolic profile (> 20% change) are shown. **Figure S7.** Metabotranscriptomic analysis of microarray and metabolite profile in cells treated with 1.0 µg/µl MNPs@SiO_2_(RITC) for 12 h. Lipid peroxidation and focal adhesion related genes and metabolites network were constructed algorithmically by IPA in 1.0 µg/µl MNPs@SiO_2_(RITC)-treated HEK293 cells. Red and green areas indicate up- and downregulated genes, respectively. Orange and blue colours indicate activation and suppression, respectively. The lines indicate indirect (dotted) or direct (solid) relationship. Differentially expressed genes obtained from microarray data (> 3-fold change) and disturbances in metabolic profile (> 20% change) are shown. **Figure S8.** Z-stack analysis for NPs treated HEK293 cells. The cells were treated with silica NPs and MNPs@SiO_2_(RITC) for 12 h. The locations of NPs were analysed with *z*-stack mode of confocal microscopy. Nucleus, blue; NPs, red; ubiquitin, green; lysosome, violet. Scale bar= 10 μm.

**Additional file 2: Movie S1.** Tracking movement of non-treated control HEK293 cells on pillars for 24 h.

**Additional file 3: Movie S2.** Tracking movement of 0.1 μg/μL MNPs@SiO_2_(RITC) treated HEK293 cells on pillars for 24 h.

**Additional file 4: Movie S3.** Tracking movement of 1.0 μg/μL MNPs@SiO_2_(RITC) treated HEK293 cells on pillars for 24 h.
