# Supplementary material for: Decrease in membrane fluidity and traction force induced by silica-coated magnetic nanoparticles
Source: J Nanobiotechnology. 2021 Jan 11;19:21. doi: 10.1186/s12951-020-00765-5 (PMC7802323; doi:10.1186/s12951-020-00765-5)
Supplement: Supplementary file 1 — Additional file 1: Table S1. Ingenuity Pathway Analysis-based profiles of transcriptomic network-related genes in MNPs@SiO2(RITC)-treated cells. Table S2. RT-PCR and real-time PCR primer sequences for genes encoding transcriptomic network-related genes. Figure S1. Determination of size and homogeneity for MNPs@SiO2(RITC) and silica NPs using transmission electron microscope (TEM) analysis. TEM images of MNPs@SiO2(RITC) (a) and silica NPs (b). Scale bar = 50 nm. Figure S2. Evaluation of cytotoxicity in silica NPs and MNPs@SiO2(RITC) treated HEK293 cells. HEK293 cells were treated with silica NPs and MNPs@SiO2(RITC) for 12 h. The changes in cell viability were evaluated with MTS. Data represent mean ± SD of three independent experiments. N.S: Not significant. Figure S3. Low magnification laurdan GP images. Merged DIC and TIRFM images (upper panel) of HEK293 cells. Distributions of MNPs@SiO2(RITC) are shown in each lower panel. GP distributions ranged from −1.0 to 1.0. Scale bar= 2.5 µm. Figure S4. ROS generation in silica NPs and MNPs@SiO2(RITC)-treated cells. Evaluation of intracellular ROS generation using DCFH-DA after 12 h silica NPs and MNPs@SiO2(RITC) treatment in HEK293 cells. The intensity of non-oxidised DCFH-DA was used as a blank. Data represent mean ± SD of three independent experiments. *p < 0.05 vs non-treated control, #p < 0.05 compared between 0.1 and 1.0 µg/µl of NPs-treated cells. N.S: Not significant. Figure S5. Transcriptomic analysis of microarray in HEK293 cells treated with MNPs@SiO2(RITC) for 12 h. Network of lipid peroxidation and focal adhesion related genes was constructed algorithmically by IPA. (a) Transcriptome network of 0.1 µg/µl MNPs@SiO2(RITC)-treated cells and (b) prediction analysis for the network. Red and green areas indicate up- and downregulated genes, respectively. Orange and blue colours indicate activation and suppression. The lines indicate indirect (dotted) or direct (solid) relationship. Differentially expressed genes obta [file 12951_2020_765_MOESM1_ESM.docx]

**Additional File 1-Supporting Information**

Decrease in Membrane Fluidity and Traction Force Induced by Silica-Coated Magnetic Nanoparticles

Tae Hwan Shin^1,#^, Abdurazak Aman Ketebo^2,#^_,_ Da Yeon Lee^1^, Seungah Lee^3^, Seong Ho Kang^3^, Shaherin Basith^1^, Balachandran Manavalan^1^, Do Hyeon Kwon^4^, Sungsu Park^2,*^, and Gwang Lee^1,4,*^

^1^*Department of Physiology, Ajou University School of Medicine, Suwon, Republic of Korea*

^2^*School of Mechanical Engineering, Sungkyunkwan University, Suwon, Republic of Korea*

^3^*Department of Applied Chemistry and Institute of Natural Sciences, Kyung Hee University, Yongin-si, Republic of Korea*

^4^*Department of Molecular Science and Technology, Ajou University, Suwon, Republic of Korea*

^#^These authors contributed equally to this work.

^*^Corresponding authors:

Sungsu Park, Professor, School of Mechanical Engineering, Sungkyunkwan University, Seobu-ro, Jangan-gu, Suwon-si, Gyeonggi-do, 16419, Republic of Korea. Phone: +82-31-290-7431, fax: +82-31-290-5889, e-mail: nanopark@skku.edu

Gwang Lee, Professor, Department of Physiology, Ajou University School of Medicine, 164, World cup-ro, Yeongtong-gu, Suwon-si, Gyeonggi-do 16499, Republic of Korea. Phone: +82-31-219-4554, fax: +82-31-219-5049, e-mail: glee@ajou.ac.kr

**Materials and Methods**

**Cell culture.** Human embryonic kidney 293 (HEK293) cells were obtained from American Type Culture Collection (ATCC). Briefly, the cells were cultured in Dulbecco’s high-glucose modified Eagle’s medium (DMEM, Gibco, USA) supplemented with 10% fetal bovine serum (Gibco, USA), 100 units/ml penicillin, and 100 µg/ml streptomycin (Gibco, USA) and incubated in a 5% CO_2_ humidified chamber at 37°C.

**Cell viability assay.** To analyse cytotoxicity of MNPs@SiO_2_(RITC) and silica NPs, the CellTiter 96-cell proliferation assay kit (MTS, Promega, USA) was used according to the manufacturer’s instructions. Briefly, 3 × 10^4^ HEK293 cells were seeded on a 96-well assay plate. After overnight incubation, HEK293 cells were washed with PBS and treated with MNPs@SiO_2_(RITC). After 12 h, excessive MNPs@SiO_2_(RITC) was washed with PBS. MTS solution was added to each well and the plate was incubated for 1 h in a 5% CO_2_, 37°C chamber. The plate was measured using a plate reader (Molecular Devices, USA) at 490 nm wavelength.

**Evaluation of intracellular ROS levels.** Intracellular ROS levels were evaluated by DCFH-DA staining (Cell Biolabs, San Diego, CA, USA) according to the manufacturer’s protocol. Briefly, HEK293 cells were treated with MNPs@SiO_2_(RITC) and silica NPs for 12 h. The cells were resuspended in a dye solution (10 µM DCFH-DA in PBS) and incubated at 37°C/5% CO_2_ for 1 h. Samples were washed twice with PBS, and fluorescence was measured with a Gemini EM fluorescence microplate reader (Molecular Devices, Sunnyvale, CA, USA) at 480 nm excitation/530 nm emission.

**Evaluation of lipid peroxidation.** Peroxidised unsaturated lipids were quantified according to the manufacturer’s instructions with lipid peroxidation kit (Cayman Chemical, Ann Arbor, MI, USA). Briefly, for 12 h, 0.1 and 1.0 µg/µl of MNPs@SiO_2_(RITC)-treated cells and non-treated control cells were detached from the culture dish and washed with PBS twice. The cells were transferred to glass test tubes. Lipids were extracted with methanol, which is saturated with crystalline solid and ice-cold chloroform. After centrifugation (1500g, 0^o^C, 5-times), bottom chloroform layers were collected. The collected samples were 9:1 mixed with 2.25 mM ferrous sulfate, 0.1 M hydrochloric acid, and 1.5% ammonium thiocyanate in methanol and incubated at room temperature for 5 mins. Ferric ions were produced in the reaction with the mixture, and peroxidised unsaturated lipids were detected using thiocyanate as chromogen (absorbance at 500 nm). Absorbance were measured using quartz cuvette and microplate reader (Molecular Devices, USA).

**Reverse transcription PCR (RT-PCR) analysis.** For semi-quantitative RT-PCR, a cDNA library was synthesised using iScript Advanced cDNA Synthesis Kit (Bio-rad, CA). Reaction conditions were as follows: 46°C for 20 mins, followed by 95°C for 1 min. cDNA were reverse transcribed and amplified using gene-specific primer pairs for genes related to transcriptomic network (Supplementary Table 2). To remove template errors, PCR products were normalised relative to glyceraldehyde-3-phosphate dehydrogenase (*GAPDH*), and PCR products were amplified from the same cDNA. Amplified PCR products were separated on a 2% agarose gel and stained with ethidium bromide.

**Quantitative PCR analysis.** The expression of transcriptomic network-related genes was detected by qPCR using the SYBR Green-based real-time PCR kit (Bio-Rad) with gene-specific primer pairs (Supplementary Table 2) on a Rotor Gene-Q system (Qiagen, Valencia, CA, USA). Reaction conditions were as follows: 95°C for 5 mins, followed by 50 cycles of 95°C for 5 s and 60°C for 30 s. The threshold/quantification cycle (Ct/Cq) value was determined at a point where the detected fluorescence was statistically higher than the background level. PCR products were analysed based on a melting curve constructed using Rotor-Gene 1.7 software (Qiagen). PCR reactions were prepared as independent triplicate samples. The relative quantification of target gene expression was calculated by the 2^−ΔΔCt^ method.

**Measurement of Adenosine Triphosphate (ATP) concentration.** ATP concentration of MNPs@SiO_2_(RITC)-treated HEK293 cells was measured using an ATP assay system (Promega, USA) according to the manufacturer's protocol. Briefly, HEK293 cells were seeded (3 × 10^4^) on a 96 well plate and treated with MNPs@SiO_2_(RITC) for 6, 12, and 24 h. HEK293 cells were trypsinised and counted. Luciferin reagent with cell lysis agent was mixed (1 × 10^4^ cells). The mixtures were resuspended and then the cells were split in 384-well white plates. After 20 mins of incubation at room temperature, luminescence of each well was measured with luminometer (LMaxII^384^; Molecular Devices, USA). Luminescence was recorded using a 0.3-1-s integration on a Synergy 2 luminometer (BioTek, CA) and subsequently captured using a ChemiDoc™ Touch Gel Imaging System (Bio-Rad).

**Immunocytochemistry.** HEK293 cells were seeded on cover slips and treated with 0.1 μg/μl and 1.0 μg/μl of MNPs@SiO_2_(RITC) and silica NPs for 12 h. The cells were then fixed in Cytofix buffer (BD) for 30 mins at 4^o^C. For reducing non-specific binding, cover slips were blocked with PBS containing 2% BSA and 0.1% Triton-X100 (Sigma-Aldrich). For labeling lysosome and ubiquitin, cells were incubated with lysotracker (Thermo Fisher, 10 nM) in PBS for 1h at room temperature and then anti-ubiquitin rabbit polyclonal antibody (Santa Cruz, 1:200) diluted in buffer was used for blocking at 4^o^C for 12 h. The cover slips were washed thrice with PBS containing 0.1% Triton-X100 and incubated with Alexa Fluor 488-conjugated goat anti-rabbit polyclonal antibody (Thermo Fisher, 1:200) for 2 h at room temperature. Labelled cells were washed thrice with PBS containing 0.1% Triton-X100 and incubated with PBS containing 10 µg/ ml concentration of Hoechst 33342 for 15 mins at room temperature for nucleus labelling. After washing thrice with PBS, cover slips were mounted onto slides using Prolong Gold Antifade mounting medium (Molecular Probes). Fluorescent z-stack images were acquired by confocal laser scanning microscopy (Nikon A1R, Nikon, Japan).

Table S1. Ingenuity Pathway Analysis-based profiles of transcriptomic network-related genes in MNPs@SiO_2_(RITC)-treated cells

| Entrez gene name | Symbol | Affymetrix ID | Location | Signal fold change^a^ | |
| --- | --- | --- | --- | --- | --- |
|  |  |  |  | 0.1 µg/µl | 1.0 µg/µl |
| acetylcholinesterase (Cartwright blood group) | *ACHE* | 205377_s_at | Plasma Membrane | -10.4 | -5.5 |
| A-kinase anchoring protein 12 | *AKAP12* | 1555395_at | Cytoplasm | 8.3 | 3.3 |
| apolipoprotein B | *APOB* | 223579_s_at | Extracellular Space | -3.7 | -14.1 |
| apolipoprotein E | *APOE* | 203382_s_at | Extracellular Space | 3.4 | 5.1 |
| BCL2, apoptosis regulator | *BCL2* | 203684_s_at | Cytoplasm | -7.3 | -10.5 |
| complement C3a receptor 1 | *C3AR1* | 209906_at | Plasma Membrane | -1.1 | -5.7 |
| Catalase | *CAT* | 215573_at | Cytoplasm | 13.4 | 17.4 |
| CD44 molecule (Indian blood group) | *CD44* | 234411_x_at | Plasma Membrane | -4.9 | 11.4 |
| CDC42 binding protein kinase alpha | *CDC42BPA* | 214464_at | Cytoplasm | 1.3 | 5.2 |
| complement C3d receptor 2 | *CR2* | 244097_at | Plasma Membrane | 13.0 | 6.9 |
| CRK like proto-oncogene, adaptor protein | *CRKL* | 206184_at | Cytoplasm | -1.4 | -3.6 |
| Cortactin | *CTTN* | 1567277_at | Plasma Membrane | 4.3 | 4.6 |
| fibroblast growth factor receptor 2 | *FGFR2* | 240913_at | Plasma Membrane | 6.0 | 17.0 |
| fibronectin 1 | *FN1* | 214702_at | Extracellular Space | 1.9 | -5.0 |
| integrin subunit beta 1 | *ITGB1* | 216190_x_at | Plasma Membrane | -19.4 | 29.3 |
| integrin subunit beta 1 binding protein 1 | *ITGB1BP1* | 228227_at | Plasma Membrane | -5.3 | -9.2 |
| jagged 1 | *JAG1* | 229924_s_at | Extracellular Space | -4.3 | -3.1 |
| kinase insert domain receptor | *KDR* | 203934_at | Plasma Membrane | 8.7 | 7.4 |
| Klotho | *KL* | 205978_at | Extracellular Space | 22.0 | 5.3 |
| low density lipoprotein receptor | *LDLR* | 217103_at | Plasma Membrane | -2.7 | -3.3 |
| LIM zinc finger domain containing 1 | *LIMS1* | 1566129_at | Plasma Membrane | 12.4 | 29.4 |
| microtubule associated protein tau | *MAPT* | 203930_s_at | Plasma Membrane | -1.4 | -8.3 |
| MCF.2 cell line derived transforming sequence | *MCF2* | 208017_s_at | Cytoplasm | 8.3 | 7.2 |
| NCK adaptor protein 1 | *NCK1* | 229895_s_at | Cytoplasm | -1.8 | -8.5 |
| nuclear factor, erythroid 2 like 2 | *NFE2L2* | 1567013_at | Nucleus | 2.3 | 6.8 |
| nitric oxide synthase 1 | *NOS1* | 239132_at | Cytoplasm | 5.5 | 13.1 |
| nitric oxide synthase 2 | *NOS2* | 210037_s_at | Cytoplasm | 1.2 | -16.6 |
| NADPH oxidase 4 | *NOX4* | 236843_at | Cytoplasm | -8.5 | 6.2 |
| 3-phosphoinositide dependent protein kinase 1 | *PDPK1* | 244630_at | Cytoplasm | -1.3 | -3.5 |
| pseudopodium enriched atypical kinase 1 | *PEAK1* | 234036_x_at | Plasma Membrane | 3.4 | 11.8 |
| paraoxonase 1 | *PON1* | 206344_at | Extracellular Space | -11.1 | -3.8 |
| peroxisome proliferator activated receptor alpha | *PPARA* | 1560981_a_at | Nucleus | 6.6 | -4.8 |
| protein kinase C alpha | *PRKCA* | 215195_at | Cytoplasm | 2.8 | 3.5 |
| protein kinase C epsilon | *PRKCE* | 234089_at | Cytoplasm | 17.4 | 12.6 |
| protein kinase C iota | *PRKCI* | 209678_s_at | Cytoplasm | 1.0 | 3.3 |
| presenilin 1 | *PSEN1* | 1559206_at | Plasma Membrane | 7.3 | 12.4 |
| prostaglandin E receptor 3 | *PTGER3* | 210833_at | Plasma Membrane | -78.4 | -17.8 |
| prostaglandin E receptor 4 | *PTGER4* | 204896_s_at | Plasma Membrane | -3.5 | -3.7 |
| protein tyrosine kinase 2 beta | *PTK2B* | 203111_s_at | Cytoplasm | 1.2 | 7.6 |
| protein tyrosine phosphatase, non-receptor type 1 | *PTPN1* | 217689_at | Cytoplasm | -2.5 | 3.1 |
| protein tyrosine phosphatase, non-receptor type 11 | *PTPN11* | 205867_at | Cytoplasm | -1.9 | -17.6 |
| protein tyrosine phosphatase, receptor type J | *PTPRJ* | 214137_at | Plasma Membrane | 5.1 | 4.7 |
| Paxillin | *PXN* | 211823_s_at | Cytoplasm | 1.1 | -4.0 |
| ras homolog family member A | *RHOA* | 240337_at | Cytoplasm | 18.1 | 8.5 |
| ras homolog family member B | *RHOB* | 1553963_at | Cytoplasm | -1.6 | 6.6 |
| sphingosine-1-phosphate receptor 1 | *S1PR1* | 204642_at | Plasma Membrane | -1.0 | -9.8 |
| solute carrier family 9 member A1 | *SLC9A1* | 1554728_at | Plasma Membrane | 1.7 | 7.8 |
| SMAD family member 3 | *SMAD3* | 205397_x_at | Nucleus | 1.1 | 5.0 |
| synuclein alpha | *SNCA* | 236081_at | Cytoplasm | -1.9 | 3.2 |
| superoxide dismutase 2 | *SOD2* | 215078_at | Cytoplasm | 7.3 | 13.6 |
| transforming growth factor beta 1 | *TGFB1* | 203084_at | Extracellular Space | 3.2 | 12.0 |
| transglutaminase 2 | *TGM2* | 201042_at | Cytoplasm | -1.3 | -3.3 |

^a^Normalised signal fold change of signal in treated groups with MNPs@SiO_2_(RITC) to corresponding signal in non-treated control group.

Table S2. RT-PCR and real-time PCR primer sequences for genes encoding transcriptomic network-related genes

| Gene Name | Symbol | NCBI Ref. seq | Direction | Primer sequence (5’-3’) |
| --- | --- | --- | --- | --- |
| Homo sapiens superoxide dismutase 2 | *SOD2* | BC016934.1 | Forward | TGA GCC ACA TTC CGT TAC AC |
|  |  |  | Reverse | GAC CAA ACA TTT CCC CAA AGC |
| Homo sapiens LIM zinc finger domain containing 1 | *LIMS1* | NM_001193485 | Forward | TCA AGA ATG CTG GGA GAC AC |
|  |  |  | Reverse | GAA ATG GTC TGG ATG GTA GGG |
| Homo sapiens NCK adaptor protein 1 | *NCK1* | NM_006153 | Forward | GAA CCA TCA CCT CCA CAG TG |
|  |  |  | Reverse | AAT CCC CTT CAT GTC CTC TTT C |
| Homo sapiens complement component 3a receptor 1 | *C3AR1* | BC020742 | Forward | AGG ATG AAG TGA TAT GGT GAG C |
|  |  |  | Reverse | GGA TGC GGC TTG AGA ATT TG |
| Homo sapiens glyceraldehyde 3-phosphate dehydrogenase | *GAPDH* | NM_002046 | Forward | GAA GAC TGT GGA TGG CCC |
|  |  |  | Reverse | CCA TGC CAG TGA GCT TCC |

Ref. seq.: Reference sequence


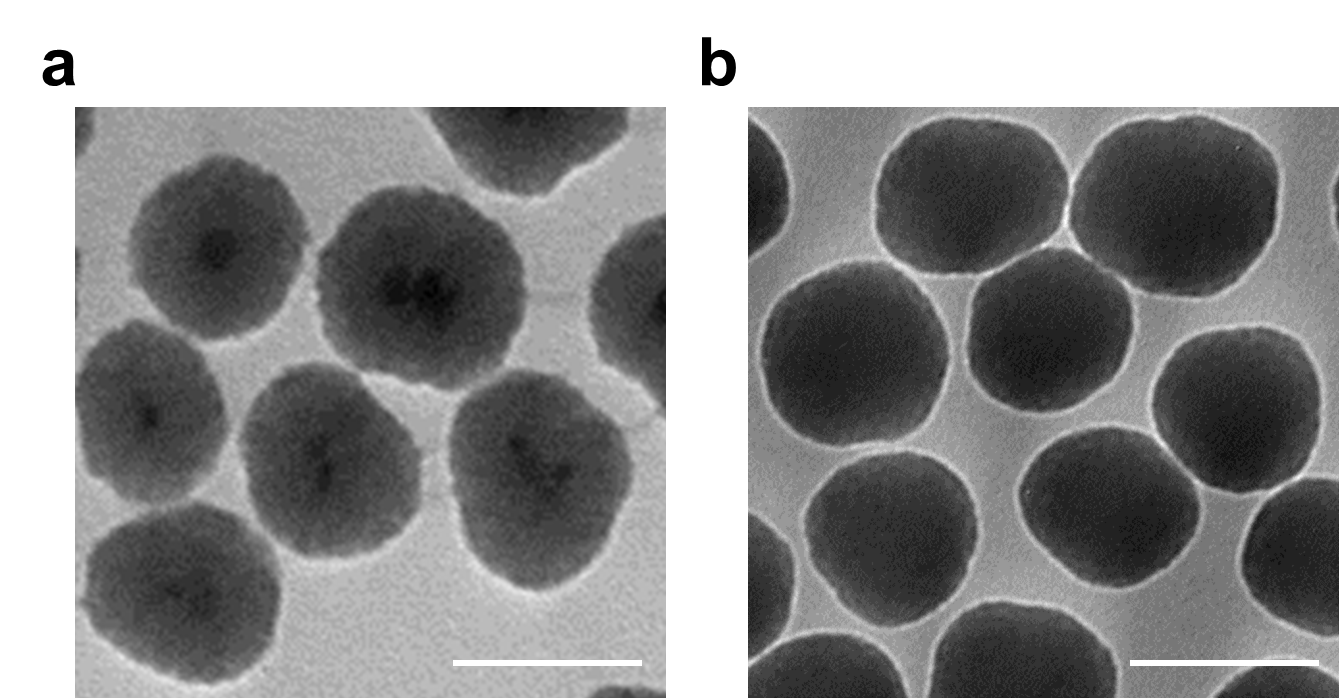


Figure S1. Determination of size and homogeneity for MNPs@SiO_2_(RITC) and silica NPs using transmission electron microscope (TEM) analysis. TEM images of **a** MNPs@SiO_2_(RITC) and **b** silica NPs. Scale bar = 50 nm.


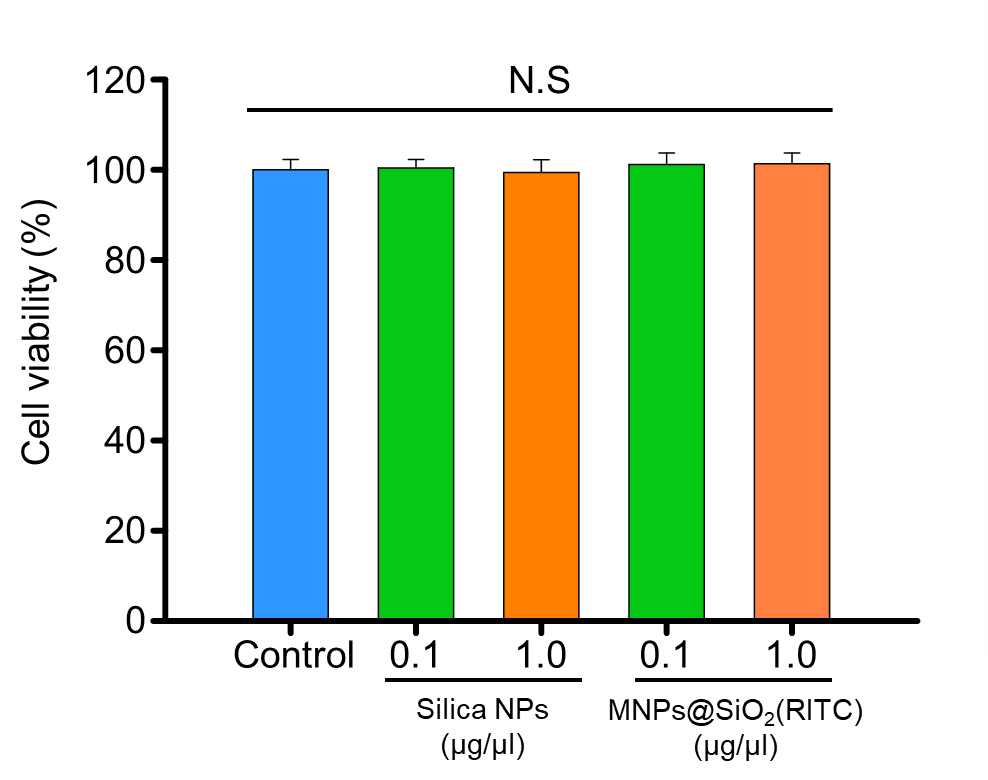


Figure S2. Evaluation of cytotoxicity in silica NPs and MNPs@SiO_2_(RITC) treated HEK293 cells. HEK293 cells were treated with silica NPs and MNPs@SiO_2_(RITC) for 12 h. The changes in cell viability were evaluated with MTS. Data represent mean ± SD of three independent experiments. N.S: Not significant.


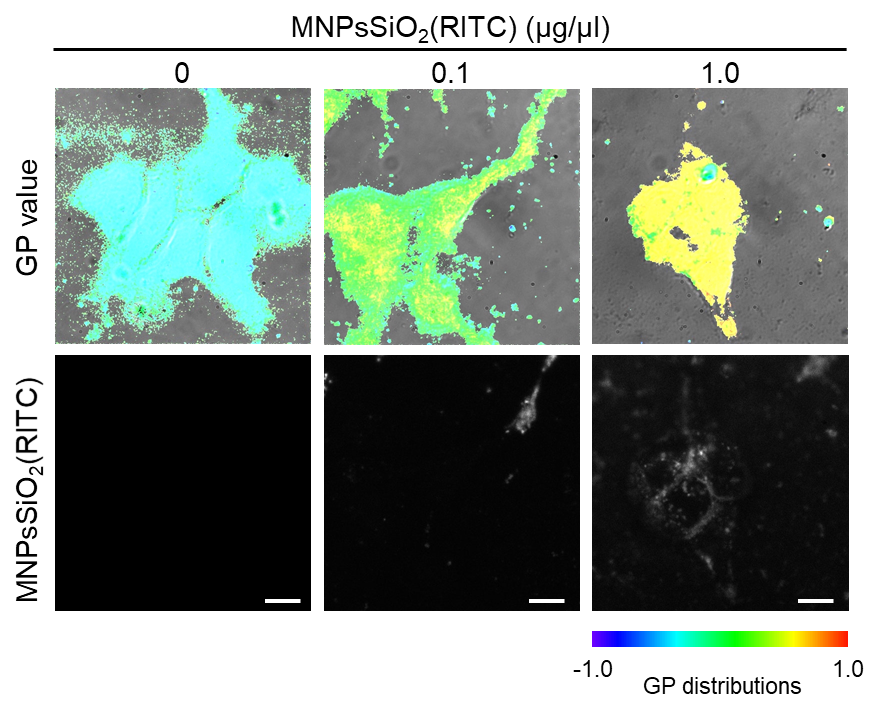


Figure S3. Low magnification laurdan GP images. Merged DIC and TIRFM images (upper panel) of HEK293 cells. Distributions of MNPs@SiO_2_(RITC) are shown in each lower panel. GP distributions ranged from −1.0 to 1.0. Scale bar= 2.5 µm.


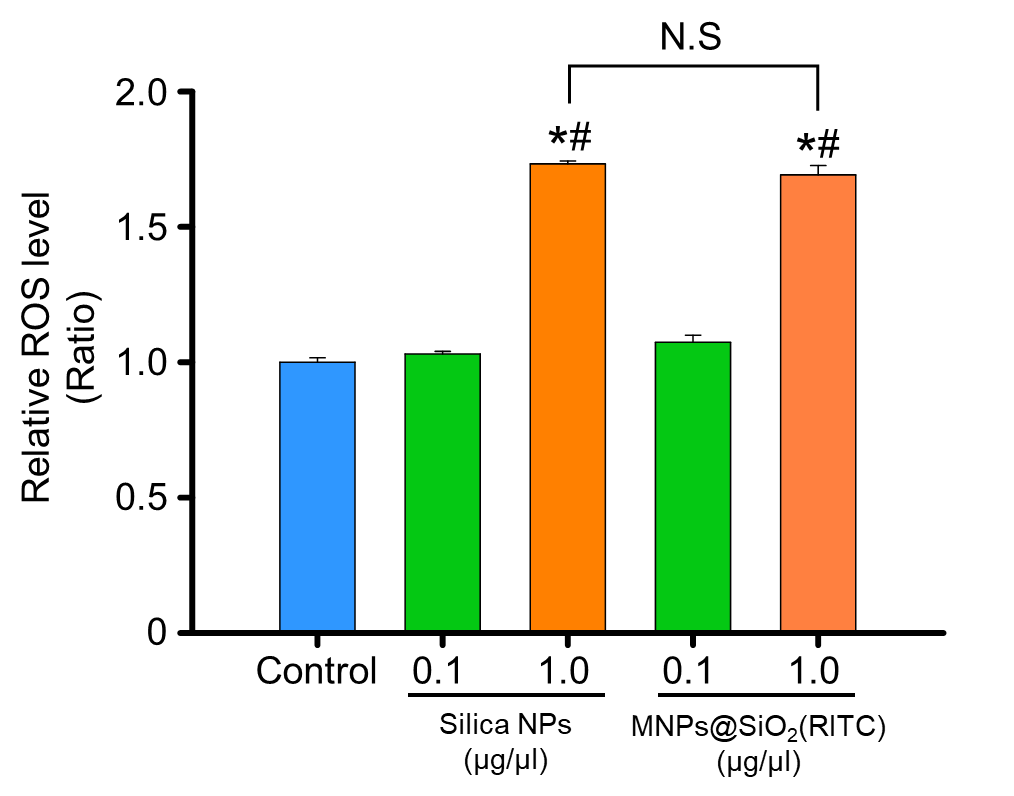


Figure S4. ROS generation in silica NPs and MNPs@SiO_2_(RITC)-treated cells. Evaluation of intracellular ROS generation using DCFH-DA after 12 h silica NPs and MNPs@SiO_2_(RITC) treatment in HEK293 cells. The intensity of non-oxidised DCFH-DA was used as a blank. Data represent mean ± SD of three independent experiments. **p <* 0.05 *vs* non-treated control, ^#^*p <* 0.05 compared between 0.1 and 1.0 µg/µl of NPs-treated cells. N.S: Not significant.


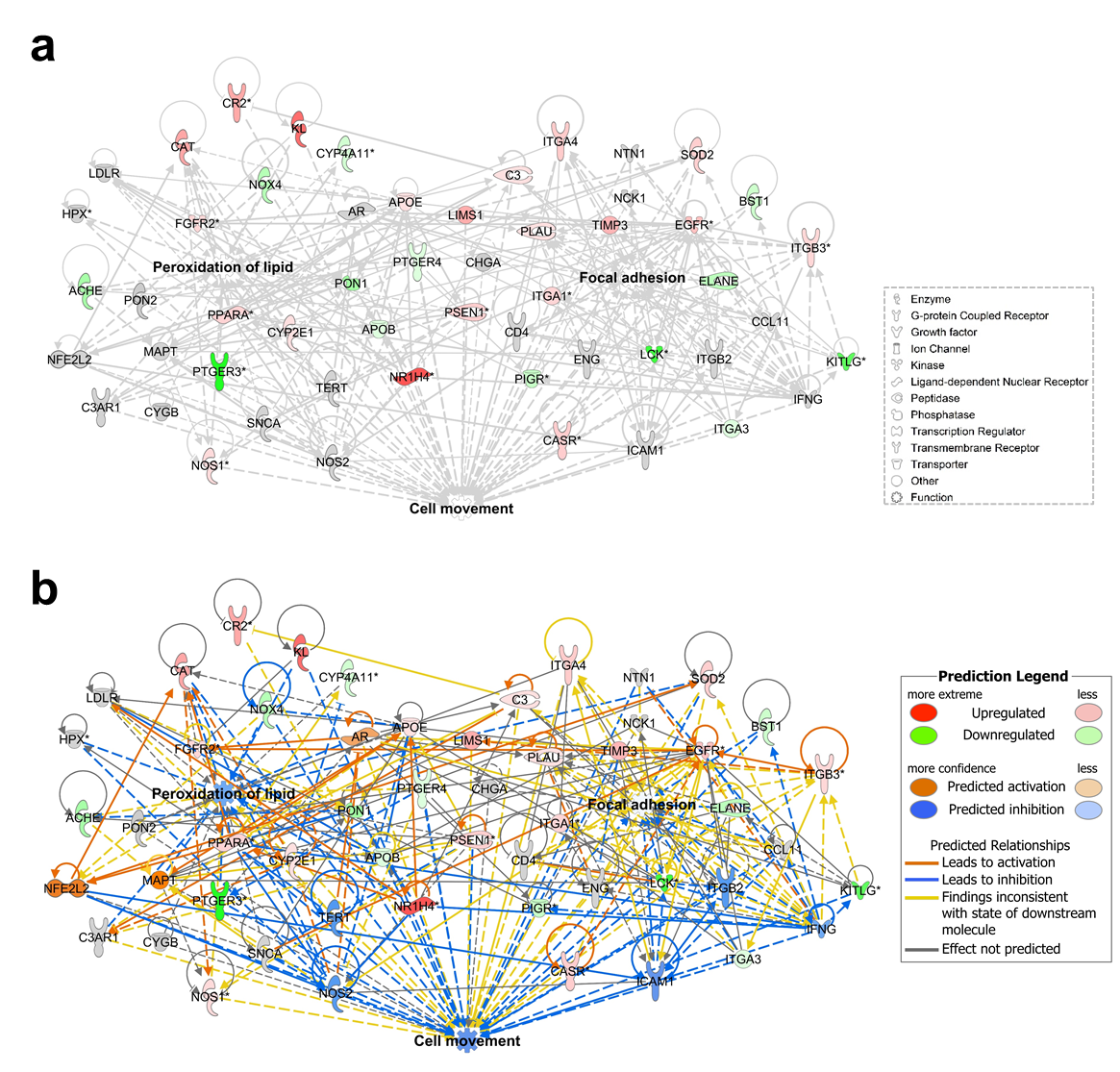


Figure S5. Transcriptomic analysis of microarray in HEK293 cells treated with MNPs@SiO_2_(RITC) for 12 h. Network of lipid peroxidation and focal adhesion related genes was constructed algorithmically by IPA. **a** Transcriptome network of 0.1 µg/µl MNPs@SiO_2_(RITC)-treated cells and **b** prediction analysis for the network. Red and green areas indicate up- and downregulated genes, respectively. Orange and blue colours indicate activation and suppression. The lines indicate indirect (dotted) or direct (solid) relationship. Differentially expressed genes obtained from microarray data (genes with > 3-fold change) are shown.


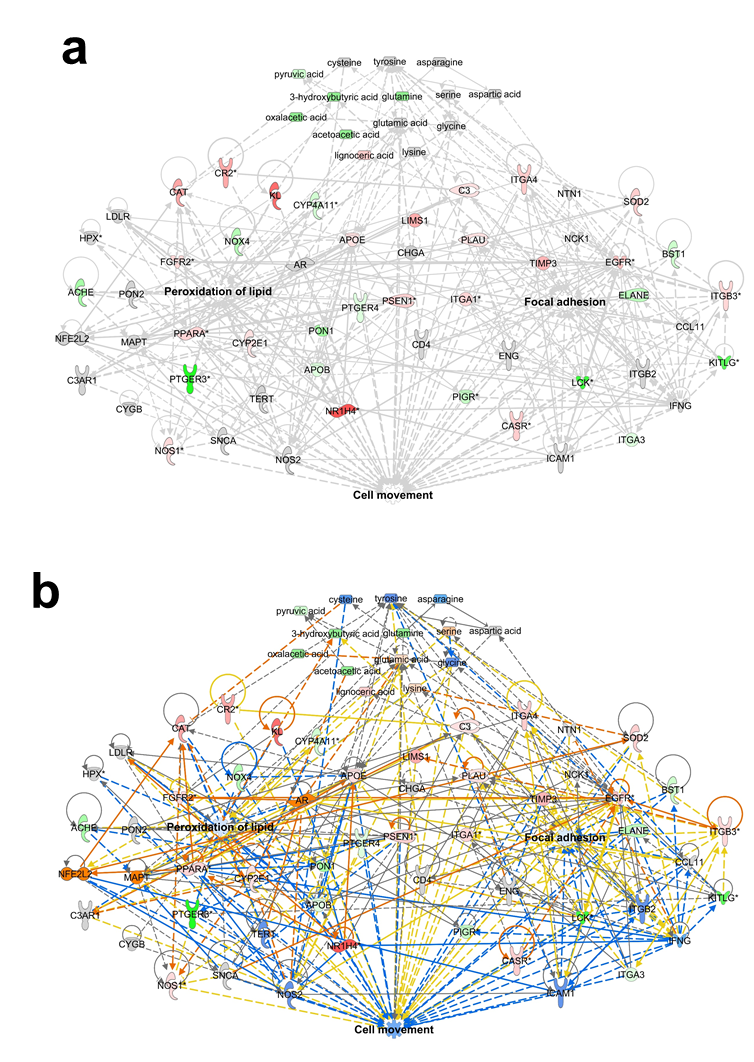


Figure S6. Metabotranscriptomic analysis of microarray and metabolite profiles in cells treated with 0.1 µg/µl MNPs@SiO_2_(RITC) for 12 h. **a** Lipid peroxidation and focal adhesion related genes and metabolites network were constructed algorithmically by IPA in 0.1 µg/µl MNPs@SiO_2_(RITC)-treated HEK293 cells and **b** prediction analysis for the network. Red and green areas indicate up- and downregulated genes, respectively. Orange and blue colours indicate activation and suppression, respectively. The lines indicate indirect (dotted) or direct (solid) relationship. Differentially expressed genes obtained from microarray data (> 3-fold change) and disturbances in metabolic profile (> 20% change) are shown.


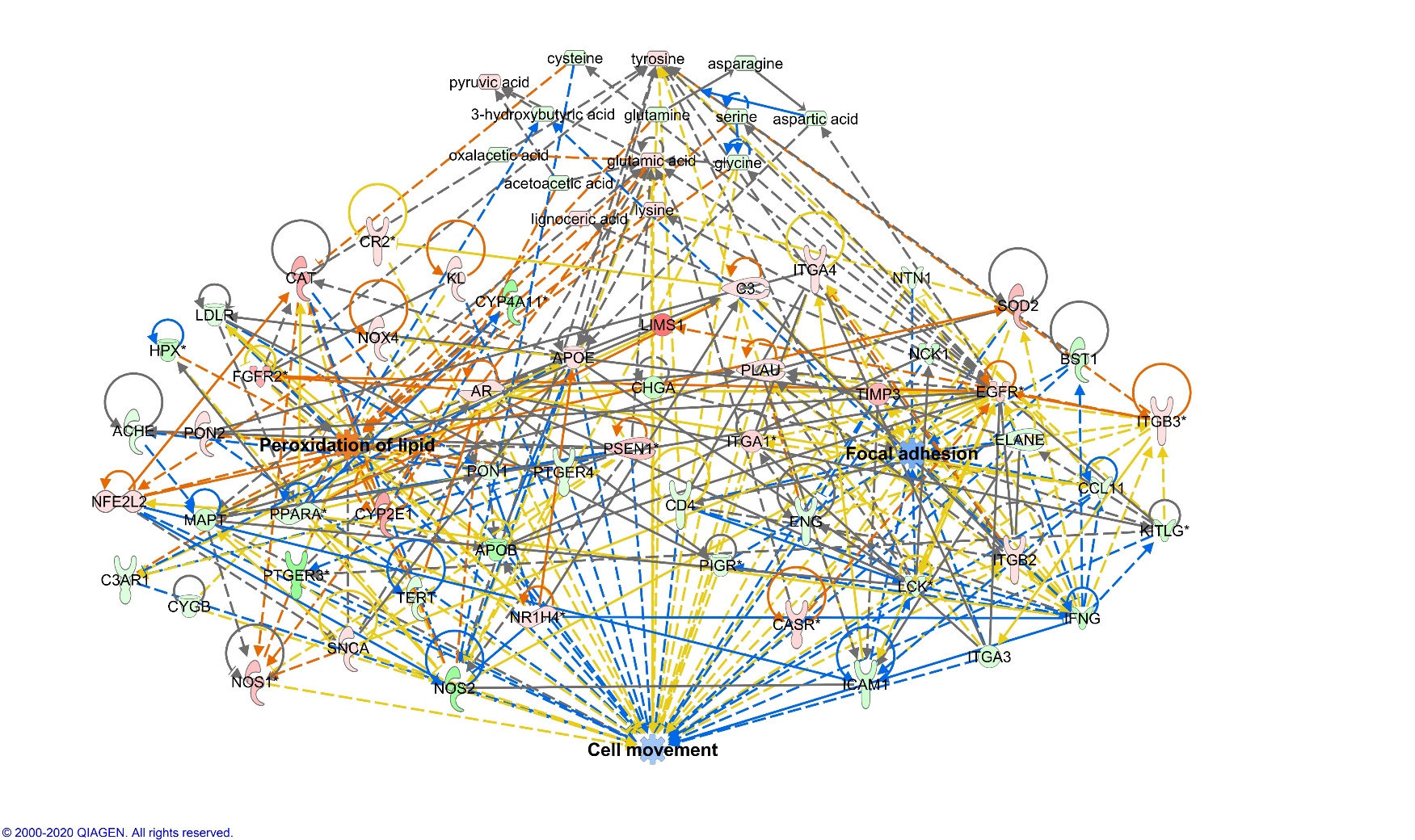


Figure S7. Metabotranscriptomic analysis of microarray and metabolite profile in cells treated with 1.0 µg/µl MNPs@SiO_2_(RITC) for 12 h. Lipid peroxidation and focal adhesion related genes and metabolites network were constructed algorithmically by IPA in 1.0 µg/µl MNPs@SiO_2_(RITC)-treated HEK293 cells. Red and green areas indicate up- and downregulated genes, respectively. Orange and blue colours indicate activation and suppression, respectively. The lines indicate indirect (dotted) or direct (solid) relationship. Differentially expressed genes obtained from microarray data (> 3-fold change) and disturbances in metabolic profile (> 20% change) are shown.


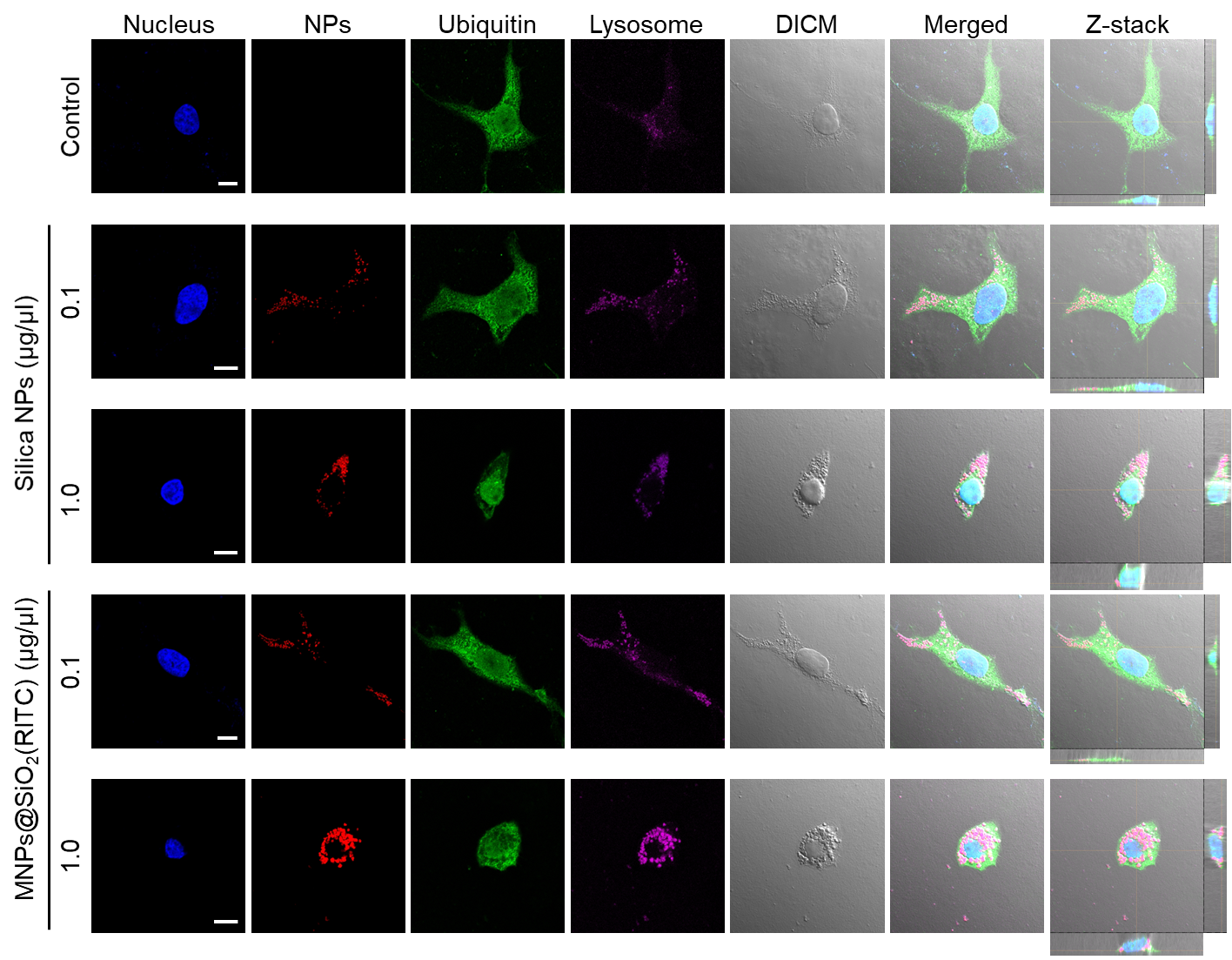


Figure S8. Z-stack analysis for NPs treated HEK293 cells. The cells were treated with silica NPs and MNPs@SiO_2_(RITC) for 12 h. The locations of NPs were analysed with *z*-stack mode of confocal microscopy. Nucleus, blue; NPs, red; ubiquitin, green; lysosome, violet. Scale bar= 10 μm.
